# SAIGE-GPU: accelerating genome- and phenome-wide association studies using GPUs

**DOI:** 10.1093/bioinformatics/btag032

**Published:** 2026-01-22

**Authors:** Alex Rodriguez, Youngdae Kim, Tarak Nath Nandi, Karl Keat, Rachit Kumar, Mitchell Conery, Rohan Bhukar, Molei Liu, John Hessington, Ketan Maheshwari, VA Million Veteran Program, Sumitra Muralidhar, Jennifer Moser, Jennifer E Deen, Philip S Tsao, Sumitra Muralidhar, J Michael Gaziano, Elizabeth Hauser, Amy Kilbourne, Michael Matheny, Dave Oslin, J Michael Gaziano, Jessica V Brewer, Mary T Brophy, Kelly Cho, Lori Churby, Scott L DuVall, Saiju Pyarajan, Luis E Selva, Shahpoor (Alex) Shayan, Stacey B Whitbourne, J Michael Gaziano, Brady Stephens, Todd Connor, Themistocles L Assimes, Adriana Hung, Henry Kranzler, Samuel Aguayo, Sunil Ahuja, Kathrina Alexander, Xiao M Androulakis, Prakash Balasubramanian, Zuhair Ballas, Elizabeth S Bast, Jean Beckham, Sujata Bhushan, Edward Boyko, David Cohen, Louis Dellitalia, Gerald Wayne Dryden, L Christine Faulk, Joseph Fayad, Daryl Fujii, Saib Gappy, Frank Gesek, Michael Godschalk, Jennifer Greco, Todd W Gress, Samir Gupta, Salvador Gutierrez, Mark Hamner, John Harley, Daniel J Hogan, Adriana Hung, Robin Hurley, Pran Iruvanti, Frank Jacono, Darshana Jhala, Seema Joshi, Scott Kinlay, Michael Landry, Peter Liang, Suthat Liangpunsakul, Jack Lichy, Tze Shien Lo, C Scott Mahan, Ronnie Marrache, Stephen Mastorides, Kristin Mattocks, Paul Meyer, Jonathan Moorman, Providencia Morales, Timothy Morgan, Maureen Murdoch, Eknath Naik, James Norton, Olaoluwa Okusaga, Michael K Ong, Kris Ann Oursler, Ismene Petrakis, Samuel Poon, Amneet S Rai, Michael Rauchman, Richard Servatius, Satish Sharma, River Smith, Peruvemba Sriram, Patrick Strollo, Neeraj Tandon, Philip Tsao, Gerardo Villareal, Jessica Walsh, John Wells, Jeffrey Whittle, Mary Whooley, Peter Wilson, Junzhe Xu, Shing Shing Yeh, Andrew W Yen, Edmon Begoli, Georgia Tourassi, Sumitra Muralidhar, Pradeep Natarajan, Benjamin F Voight, Kelly Cho, John Michael Gaziano, Scott M Damrauer, Katherine P Liao, Wei Zhou, Jennifer E Huffman, Anurag Verma, Ravi K Madduri

**Affiliations:** Data Science and Learning, Argonne National Laboratory, Lemont, IL 60439, United States; Department of Industrial Engineering, Artificial Intelligence Graduate School, Ulsan National Institute of Science and Technology, Ulsan 44919, Republic of Korea; Data Science and Learning, Argonne National Laboratory, Lemont, IL 60439, United States; Institute for Biomedical Informatics, University of Pennsylvania – Perelman School of Medicine, Philadelphia, PA 19104, United States; Institute for Biomedical Informatics, University of Pennsylvania – Perelman School of Medicine, Philadelphia, PA 19104, United States; Data Science and Learning, Argonne National Laboratory, Lemont, IL 60439, United States; Department of Systems Pharmacology and Translational Therapeutics, University of Pennsylvania – Perelman School of Medicine, Philadelphia, PA 19104, United States; Program in Medical and Population Genetics, Cambridge, MA 02142, United States; Cardiovascular Research Center, Massachusetts General Hospital, Boston, MA 02114, United States; Department of Biostatistics, Columbia University’s Mailman School of Public Health, New York, NY 10032, United States; Information Systems, University of Pennsylvania, Philadelphia, PA 19104, United States; Oak Ridge National Laboratory, Oak Ridge, TN 37830, United States; See Supplement for a List of MVP Contributors; Oak Ridge National Laboratory, Oak Ridge, TN 37830, United States; Computing and Computational Sciences Directorate, Oak Ridge National Laboratory, Oak Ridge, TN 37830, United States; Department of Veterans Affairs, Office of Research and Development, Washington, DC 20420, United States; Cardiovascular Research Center, Massachusetts General Hospital, Boston, MA 02114, United States; Department of Medicine, Harvard Medical School, Boston, MA 02115, United States; Program in Medical and Population Genetics and Cardiovascular Disease Initiative, Broad Institute of Harvard and MIT, Cambridge, MA 02142, United States; Cardiology Division, Massachusetts General Hospital, Boston, MA 02114, United States; Department of Systems Pharmacology and Translational Therapeutics, University of Pennsylvania – Perelman School of Medicine, Philadelphia, PA 19104, United States; Corporal Michael Crescenz VA Medical Center, Philadelphia, PA 19104, United States; Department of Genetics, University of Pennsylvania – Perelman School of Medicine, Philadelphia, PA 19104, United States; Institute for Translational Medicine and Therapeutics, University of Pennsylvania – Perelman School of Medicine, Philadelphia, PA 19104, United States; Department of Medicine, Harvard Medical School, Boston, MA 02115, United States; MVP Boston Coordinating Center, VA Boston Healthcare System, Boston, MA 02111, United States; Division of Aging, Department of Medicine, Brigham and Women’s Hospital, Boston, MA 02115, United States; Department of Medicine, Harvard Medical School, Boston, MA 02115, United States; MVP Boston Coordinating Center, VA Boston Healthcare System, Boston, MA 02111, United States; Division of Aging, Department of Medicine, Brigham and Women’s Hospital, Boston, MA 02115, United States; Corporal Michael Crescenz VA Medical Center, Philadelphia, PA 19104, United States; Department of Genetics, University of Pennsylvania – Perelman School of Medicine, Philadelphia, PA 19104, United States; Department of Surgery, University of Pennsylvania – Perelman School of Medicine, Philadelphia, PA 19104, United States; Cardiovascular Institute, University of Pennsylvania – Perelman School of Medicine, Philadelphia, PA 19104, United States; Department of Medicine, Harvard Medical School, Boston, MA 02115, United States; Massachusetts Veterans Epidemiology Research and Information Center (MAVERIC), VA Boston Healthcare System, Boston, MA 02130, United States; Department of Biomedical Informatics, Harvard Medical School, Boston, MA 02115, United States; Medicine, Rheumatology, VA Boston Healthcare System, Boston, MA 02130, United States; Division of Rheumatology, Department of Medicine, Inflammation, and Immunity, Brigham and Women’s Hospital, Boston, MA 02115, United States; Program in Medical and Population Genetics, Cambridge, MA 02142, United States; Analytic and Translational Genetics Unit, Department of Medicine, Massachusetts General Hospital, Boston, MA 02114, United States; Stanley Center for Psychiatric Research, Cambridge, MA 02142, United States; Department of Medicine, Harvard Medical School, Boston, MA 02115, United States; Massachusetts Veterans Epidemiology Research and Information Center (MAVERIC), VA Boston Healthcare System, Boston, MA 02130, United States; Palo Alto Veterans Institute for Research (PAVIR), Palo Alto Health Care System, Palo Alto, CA 94304, United States; Institute for Biomedical Informatics, University of Pennsylvania – Perelman School of Medicine, Philadelphia, PA 19104, United States; Corporal Michael Crescenz VA Medical Center, Philadelphia, PA 19104, United States; Division of Translational Medicine and Human Genetics, Department of Medicine, University of Pennsylvania – Perelman School of Medicine, Philadelphia, PA 19104, United States; Data Science and Learning, Argonne National Laboratory, Lemont, IL 60439, United States

## Abstract

**Motivation:**

Genome-wide association studies (GWAS) at biobank scale are computationally intensive, especially for admixed populations requiring robust statistical models. SAIGE is a widely used method for generalized linear mixed-model GWAS but is limited by its CPU-based implementation, making phenome-wide association studies impractical for many research groups.

**Results:**

We developed SAIGE-GPU, a GPU-accelerated version of SAIGE that replaces CPU-intensive matrix operations with GPU-optimized kernels. The core innovation is distributing genetic relationship matrix calculations across GPUs and communication layers. Applied to 2068 phenotypes from 635 969 participants in the Million Veteran Program, including diverse and admixed populations, SAIGE-GPU achieved a 5-fold speedup in mixed model fitting on supercomputing infrastructure and cloud platforms. We further optimized the variant association testing step through multi-core and multi-trait parallelization. Deployed on Google Cloud Platform and Azure, the method provided substantial cost and time savings.

**Availability and implementation:**

Source code and binaries are available for download at https://github.com/saigegit/SAIGE/tree/SAIGE-GPU-1.3.3. A code snapshot is archived at Zenodo for reproducibility (DOI: [10.5281/zenodo.17642591]). SAIGE-GPU is available in a containerized format for use across HPC and cloud environments and is implemented in R/C++ and runs on Linux systems.

## 1 Introduction

Biobanks linked to electronic health records are powering large-scale genome-wide association studies (GWAS) and other translational research (Gottesman *et al.* 2013, Sudlow *et al.* 2015, Wolford *et al*. 2018, Verma *et al.* 2022, Kurki *et al.* 2023, Zawistowski *et al.* 2023, Bick *et al.* 2024). However, the size of modern cohorts presents major computational challenges. Mixed-model GWAS rely on generalized linear mixed models (GLMMs) to control for population structure and relatedness (Uffelmann *et al.* 2021), and their cost scales with both sample and number of variants (Chang *et al.* 2015). Broad phenotyping in biobanks has further driven growth of multi-trait and phenome-wide analyses, amplifying the need for faster and more scalable computations (Sakaue *et al.* 2021, Karczewski *et al.* 2024, Verma *et al*. 2024).

To meet these demands, we optimized SAIGE (Zhou *et al.* 2018), a leading mixed-model GWAS method, by integrating Graphics Processing Units (GPUs) acceleration and distributed computing. SAIGE accounts for relatedness using a genetic relationship matrix (GRM); although full GRMs offer improved control of confounding (Lee *et al.* 2012), they are computationally intensive. SAIGE uses a conjugate-gradient solver (Kaasschieter 1988) that avoids explicit GRM construction, an approach shared with methods like Bolt-LMM (Loh *et al.* 2015), and is well suited to GPU architectures with high memory bandwidth and massive parallelism (Sunitha *et al*. 2017, Baji 2018, Cook).

The computational cost of phenome-wide GWAS remains a key bottleneck preventing many research groups from conducting such analyses. In prior work, our SAIGE-GPU implementation enabled a phenome-wide GWAS in the Million Veteran Program (MVP) (Verma *et al*. 2024), analyzing 3.5 billion genetic variants, over 2000 traits, and 635 969 MVP participants while achieving a 5-fold speedup in GLMM fitting. Standard CPU-based SAIGE would have required approximately 481 186 CPU-core hours—equivalent to 55 years of single-core time or 126 days on a 16-core workstation.

Large-scale resources such as UK Biobank, All of Us, and other national cohorts make phenome-wide GWAS increasingly common, yet computational cost remains a barrier. GPU acceleration substantially reduces this burden: cloud benchmarks show that a single-trait analysis costing $3.88 on CPUs costs $1.45 on one GPU—a 2.7× reduction. For 1000 traits, this saves approximately $2430 in compute cost ($3880 versus $1450) enabling iterative analyses that would otherwise exceed typical budgets or require national supercomputing facilities.

To confirm that GPU acceleration addresses a true bottleneck rather than providing marginal improvements, we directly compared SAIGE-GPU and REGENIE v4.1 (Mbatchou *et al.* 2021)—a widely used alternative—using identical datasets and hardware (400 000 samples, 121 587 variants). SAIGE-GPU completed model fitting step in approximately 15 min using three GPUs, while REGENIE required 31–34 min using 16–32 optimized CPU threads, a 2–2.3× speedup. In a phenome-wide analysis of 2068 traits, this difference corresponds to approximately 23 days of compute saved. Although SAIGE and REGENIE use distinct statistical frameworks (exact versus approximate), these benchmarks show that GPU acceleration of SAIGE’s model fitting step—the dominant computational cost—offers practical advantages over CPU-based implementations and alternative approximate methods. Full comparative results appear in Tables 6 and 7, available as supplementary data at *Bioinformatics* online.

## 2 Materials and methods

### 2.1 Optimizations for SAIGE linear mixed model fitting (Step 1)

SAIGE fits a GLMM for each phenotype (Y) to account for fixed covariates (X), genotypes (G) and sample relatedness through random genetic effects (b). Specifically, the model uses the logistic mixed model and can be written as:


(1) $$\mathrm{logit}(\mu_{i})=\;X_{i}\alpha +G_{i}\beta +b_{i}+e$$

where, $\mu_{i}=P(y_{i}=1|X_{i},G_{i},b_{i})$, α and β are vectors of fixed-effect and genetic-effect coefficients, and b ∼ N(0,τψ). The genetic relationship matrix (GRM) ψ is defined as:


(2) $$\psi =\frac{1}{M}{\mathrm{AA}}^{T}$$

with genotype matrix $A\in R^{\mathrm{NxM}}$. Fitting the model under the null hypothesis (β  =  0) is an iterative process, and each iteration involves solving the linear system Σx=b, where $\Sigma =W^{-1}+\tau \Psi$ with W denoting the diagonal weight matrix. Constructing and storing an explicit N×N GRM becomes infeasible as sample size and variant count grow. SAIGE addresses this by never materializing the GRM; instead, it encodes genetic relatedness through efficient matrix-vector operations used within the preconditioned conjugate gradient (PCG) solver. This implicit formulation dramatically reduces memory demands and removes the need to store the full GRM.

The main computational cost is the PCG iteration. Each iteration requires two matrix-vector multiplications with A and $A^{T}$, an O(MN) operation. Empirically, it converges in $O(N^{0.5})$ iterations (Loh *et al.* 2015), giving a total complexity $O(MN^{1.5})$. Convergence depends on the eigenvalue distribution of Σ, and clustering via preconditioning can accelerate the convergence (Nocedal and Wright 2006). The current implementation uses a diagonal preconditioning. A more sophisticated preconditioning is reserved for future work.

The current version of SAIGE relies on Intel’s Threading Building Blocks (TBB) library (Reinders 2007) for CPU parallelism, which is incompatible with some architectures such as IBM POWER9. This prevented deployment of native SAIGE on the DOE Oak Ridge Summit supercomputer used for MVP analyses.

### 2.2 GPU-accelerated and distributed implementation

To overcome scaling and hardware limitations, we developed a GPU-accelerated, distributed version of SAIGE to fit the model. The genotype matrix A is column-partitioned across nodes using the Message Passing Interface (MPI) (Nielsen 2016), so node i stores $A_{:,s_{i}:e_{i}}$. During each PCG iteration, the GRM-vector product as:


(3) $$\psi v=\frac{1}{M}\sum_{i}A_{:,s_{i}:e_{i}}(A_{:,s_{i}:e_{i}}^{T}v)$$

**Figure 1:**
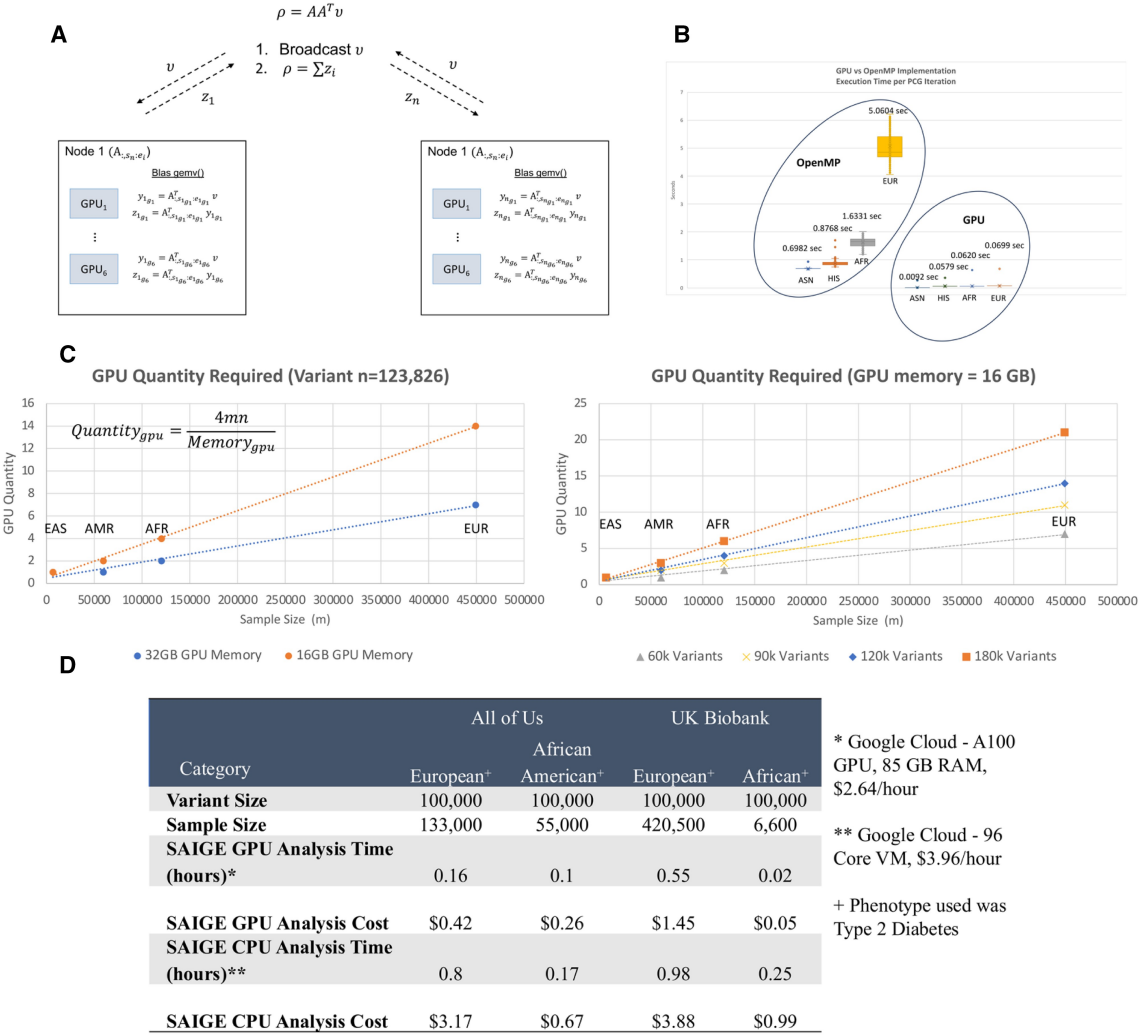
(A) Distributed BLAS *gemv()*, matrix-vector multiplication, using GPUs on the cluster. The columns of matrix A are distributed and preloaded on GPUs, with node i having columns with indices from $s_{i}$ to $e_{i}$, and these columns are distributed on GPUs on that node. The distributed matrix-vector multiplication computes $p=AA^{T}v$ through partial products on each node, avoiding explicit formation of the full GRM matrix. These partial products are aggregated to compute a solution p. (B) Demonstration of the time required for a single PCG iteration on a GPU, showcasing the efficient parallelization within the GPU. (C) GPU node requirements scale linearly with genotype matrix size (*m* samples × *n* variants). Left panel shows the effect of GPU memory size (16 GB versus 32 GB) with fixed variant count (*n* = 123 826). Right panel shows the effect of variant count with fixed GPU memory (16 GB). The relationship follows Quantity_gpu = 4 mn/Memory_gpu, where 4 is the byte size of a single-precision floating-point number and Memory_gpu is in bytes (e.g. 16 GB = 16 × 10^9^ bytes). (D) Cost and time execution comparison using All of Us and UK Biobank data on Google Cloud Platform for SAIGE-GPU version versus the native SAIGE version.

Each node performs both multiplications on its GPUs using NVIDIA’s *cuBLAS* library *cublasgemv* (clMathLibraries/clBLAS). Partial results are summed and broadcast using MPI. This hybrid GPU-MPI design reduces both runtime and memory overhead, enabling scalable mixed-model inference across heterogeneous clusters (Fig. 1A).

Because the GPUs have limited memory relative to CPUs, very large matrices often require multi-GPU distribution. The number of GPUs needed to store and process the GRM implicitly is:


(4) $$n_{\mathrm{gpu}}=\left\lceil \frac{4MN}{{\mathrm{GPU}}_{\mathrm{mem}}\times 10^{9}}\right\rceil$$

reflecting 4 bytes per floating-point value and unit conversion from bytes to gigabytes.

### 2.3 Optimizations for SAIGE associations test (Step 2)

SAIGE’s association testing includes two key corrections that ensure statistical robustness at biobank scale. Saddlepoint Approximation (SPA) (Daniels 1954) provides accurate values under extreme case-control imbalance, and optional Firth (1993) penalized likelihood mitigates small-sample bias and separation issues. Together, these corrections preserve type I error control across a wide range of allele frequencies and phenotype distributions.

Building on this, we optimized SAIGE’s second step by parallelizing both across phenotypes and within each phenotype. When the number of threads is set to >1, the sample list is split into equal chunks processed in parallel via R’s *mclapply* and UNIX multiprocessing utilities.

We added support for a trait manifest file, allowing multiple phenotypes to be processed in a single batch by listing all required GMMAT model files, variance ratio files, and output destinations. This approach maximizes CPU utilization, reduces redundant file I/O, and allows batching as many traits as memory permits.

### 2.4 Biobank-scale analyses across population groups

We applied SAIGE-GPU to a phenome-wide GWAS of the 635 969 MVP participants (Gaziano *et al.* 2016, Verma *et al*. 2024) across four population groups: African, Admixed Americans, East Asian, and European (Table 1, available as supplementary data at *Bioinformatics* online). This required 4045 individual SAIGE runs. Model fitting used LD-pruned directly genotyped variants; association testing used imputed dosages. All analyses adjusted for age, sex, and population-specific genetic principal components. In total, over 350 billion variant-trait tests were detected (Fig. 1, available as supplementary data at *Bioinformatics* online).

### 2.5 Computational infrastructures

Initial analyses were run on the OLCF Summit supercomputer, equipped with two IBM POWER9 CPUs (512 GB DDR4) and six NVIDIA V100 GPUs (96 GB HBM2). Since POWER9—used mainly in national labs—is incompatible with Intel TBB and not representative of the x86 systems common in genomics research, we developed a custom CPU baseline for comparison.

To benchmark on standard x86 architectures, we used OLCF Frontier. Each node includes an AMD EPYC processor with 56 cores, four AMD MI250X GPUs (64 GB HBM2E each; 8 logical GPUs), and 512 GB DDR4 memory. We used three physical GPUs per trait to match Summit’s memory footprint and ensure fair comparison. SAIGE v1.4.4.1 was containerized and executed using all 56 CPU cores. Docker and Singularity containers were also tested on Google Cloud Platform (GCP) and Microsoft Azure.

## 3 Results

### 3.1 GPU acceleration for SAIGE step 1 (model fitting)

Standard SAIGE was not tractable at MVP scale or deployable on OLCF’s Summit architecture, motivating development of SAIGE-GPU. In benchmarking on Summit, GPU acceleration dramatically reduced the time per PCG iterations—from ∼5.06 s on a 42-core OpenMP implementation to ∼0.069 s on GPUs (72× faster; Fig. 1B). Overall, model fitting for a representative trait completed in ∼30 min with GPUs versus 4 h 8 min with OpenMP (3× faster; Table 2, available as supplementary data at *Bioinformatics* online), and the required number of GPUs followed the predicted scaling relationship (Fig. 1C).

Performance varied with hardware. On Summit’s higher-memory 32 GB GPUs, SAIGE-GPU achieved a 5× improvement relative to projected OpenMP performance (Table 3, available as supplementary data at *Bioinformatics* online). On Frontier—where native SAIGE could use 56 CPU cores per node—SAIGE-GPU with 3 × 64 GB GPUs achieved a more modest 2× advantage (Fig. 2, available as supplementary data at *Bioinformatics* online). These comparisons highlight that relative GPU speedups depend on CPU/GPU balance, memory capacity, and system architecture.

### 3.2 SAIGE-GPU container for cloud environment

Cloud deployment dramatically lowers barriers to entry for large-scale genomic analyses. To support cloud-based biobanks (UK Biobank, All of Us), we developed a portable SAIGE-GPU container. Across Google Cloud Platform and Microsoft Azure GPU acceleration consistently outperformed CPU-based SAIGE. For example, Type 2 Diabetes model fitting in All of Us (EUR population group) completed in ∼10 min on a single GPU versus 45 min on a 64-core VM (5× faster) at substantially lower cost ($0.42 versus $3.17) (Fig. 1D, Fig. 3, available as supplementary data at *Bioinformatics* online). UK Biobank analyses showed similar gains: ∼30 min on an A100 GPU ($1.45) versus 58 min on a 96-core CPU VM ($3.88).

### 3.3 SAIGE step 2 (association testing) parallelization

We incorporated multi-core and multi-trait parallelization into Step 2 by partitioning SNPs within traits and enabling concurrent multi-trait execution. This approach scales particularly well when many traits are processed together (Table 4, available as supplementary data at *Bioinformatics* online). In a test of 15 traits for a simulated 400 000-sample cohort, parallelized SAIGE-GPU reduced runtime by ∼11.5% relative to the native implementation (Table 5, available as supplementary data at *Bioinformatics* online). Larger gains are expected once the association testing step transitions to GPU-based kernels.

### 3.4 Future optimization directions

Remaining bottlenecks derive from large ${\mathrm{AA}}^{T}$operations, not from fixed-effects design matrices (which typically have <50 covariates), so GPU acceleration of fixed-effect terms would yield only minor (<5%) improvements. We are actively developing a GPU-optimized association-testing module; full end-to-end GPU acceleration of both SAIGE steps will provide additional speedups and further expand applicability across diverse computation environments.

## 4 Conclusion

We optimized SAIGE to fully leverage GPU acceleration, transforming previously impractical phenome-wide analyses on biobank-scale data into workloads that can be completed in under a month. These improvements substantially reduce runtime and node-hour consumption, addressing a major computational barrier as whole-genome sequencing continues to expand in scale and accessibility.

There is a growing need for mixed-model methods that operate efficiently across both HPC systems and commercial cloud platforms. SAIGE-GPU meets this need by providing scalable, portable, and cost-effective GLMM-based association testing across diverse computing environments. The tool is available in both source and containerized form for deployment on GPU-enabled systems. Source code and documentation are accessible at: https://github.com/saigegit/SAIGE/tree/SAIGE-GPU-1.3.3 and https://exascale-genomics.github.io/SAIGE-GPU. A code snapshot is archived at Zenodo for reproducibility (DOI: [10.5281/zenodo.17642591]).

## Supplementary Material

btag032_Supplementary_Data

## Data Availability

The tool is available in both source and containerized form for deployment on GPU-enabled systems. Source code and documentation are accessible at: https://github.com/saigegit/SAIGE/tree/SAIGE-GPU-1.3.3 and https://exascale-genomics.github.io/SAIGE-GPU. A code snapshot is archived at Zenodo for reproducibility (DOI: [10.5281/zenodo.17642591]).
