# Supplementary material for: SAIGE-GPU: accelerating genome- and phenome-wide association studies using GPUs
Source: Bioinformatics. 2026 Jan 22;42(3):btag032. doi: 10.1093/bioinformatics/btag032 (PMC12960912; doi:10.1093/bioinformatics/btag032)
Supplement: btag032_Supplementary_Data [file btag032_supplementary_data.zip › SAIGEComputationalSupplementary.v.1.08.Bioinformatics-MajorModifications.docx]

**Supplemental Methods, Results, and Figures**

**SAIGE-GPU - Accelerating Genome- and Phenome-Wide Association Studies using GPUs**

Alex Rodriguez^†1^, Youngdae Kim^†2^, Tarak Nath Nandi^1^, Karl Keat^3^, Rachit Kumar^3^, Mitchell Conery^1,4^, Rohan Bhukar^5,6^, Molei Liu^7^, John Hessington^8^, Ketan Maheshwari^9^, *VA Million Veteran Program*^10^, Edmon Begoli^9^, Georgia Tourassi^11^, Sumitra Muralidhar^12^, Pradeep Natarajan^6,13,14,15^, Benjamin F Voight^16,4,17,18^, Kelly Cho^19,13,20^, J Michael Gaziano^19,13,20^, Scott M Damrauer^16,17,21,22^, Katherine P Liao^23,13,24,25,26^, Wei Zhou^5,27,28^, Jennifer E Huffman^23,13,29^, Anurag Verma^‡16,3,30^, Ravi K Madduri^‡1^

^1^Data Science and Learning, Argonne National Laboratory, Lemont, IL, 60439, USA

^2^Mathematics and Computer Science Division, Argonne National Laboratory, Lemont, IL, 60439, USA

^3^Institute for Biomedical Informatics, University of Pennsylvania - Perelman School of Medicine, Philadelphia, PA, 19104, USA

^4^Department of Systems Pharmacology and Translational Therapeutics, University of Pennsylvania - Perelman School of Medicine, Philadelphia, PA, 19104, USA

^5^Program in Medical and Population Genetics, Cambridge, MA, 02142, USA

^6^Cardiovascular Research Center, Massachusetts General Hospital, Boston, MA, 02114, USA

^7^Department of Biostatistics, Columbia University's Mailman School of Public Health, New York, NY, 10032, USA

^8^Information systems, University of Pennsylvania, Philadelphia, PA, 19104, USA

^9^Oak Ridge National Laboratory, Oak Ridge, TN, USA

^10^See Supplement for a list of MVP contributors
^11^Computing and Computational Sciences Directorate, Oak Ridge National Laboratory, Oak Ridge, TN, 37830, USA

^12^Office of Research and Development, Department of Veterans Affairs, Washington, DC, 20420, USA

^13^Department of Medicine, Harvard Medical School, Boston, MA, 02115, USA

^14^Program in Medical and Population Genetics and Cardiovascular Disease Initiative, Broad Institute of Harvard and MIT, Cambridge, MA, USA

^15^Cardiology Division, Massachusetts General Hospital, Boston, MA, 02114, USA

^16^Corporal Michael Crescenz VA Medical Center, Philadelphia, PA, 19104, USA

^17^Department of Genetics, University of Pennsylvania - Perelman School of Medicine, Philadelphia, PA, 19104, USA

^18^Institute for Translational Medicine and Therapeutics, University of Pennsylvania - Perelman School of Medicine, Philadelphia, PA, 19104, USA

^19^MVP Boston Coordinating Center, VA Boston Healthcare System, Boston, MA, 02111, USA

^20^Department of Medicine, Division of Aging, Brigham and Women’s Hospital, Boston, MA, 02115, USA

^21^Department of Surgery, University of Pennsylvania - Perelman School of Medicine, Philadelphia, PA, 19104, USA
^22^Cardiovascular Institute, University of Pennsylvania - Perelman School of Medicine, Philadelphia, PA, 19104, USA

^23^Massachusetts Veterans Epidemiology Research and Information Center (MAVERIC), VA Boston Healthcare System, Boston, MA, 02130, USA
^24^Department of Biomedical Informatics, Harvard Medical School, Boston, MA, 02115, USA

^25^Medicine, Rheumatology, VA Boston Healthcare System, Boston, MA, 02130, USA
^26^Department of Medicine, Division of Rheumatology, Inflammation, and Immunity, Brigham and Women’s Hospital, Boston, MA, 02115, USA
^27^Department of Medicine, Analytic and Translational Genetics Unit, Massachusetts General Hospital, Boston, MA, 02114, USA
^28^Stanley Center for Psychiatric Research, Cambridge, MA, 02142, USA
^29^Palo Alto Veterans Institute for Research (PAVIR), Palo Alto Health Care System, Palo Alto, CA, 94304, USA

^30^Department of Medicine, Division of Translational Medicine and Human Genetics, University of Pennsylvania - Perelman School of Medicine, Philadelphia, PA, 19104, USA

**Joint Authorship**

†These authors contributed equally to this work

‡These authors supervised equally to this work

*Corresponding author: Ravi K. Madduri: [madduri@anl.gov](mailto:madduri@anl.gov)

# **Supplementary Figure 1**

The Million Veteran Program (MVP) analysis involved a series of GWAS across 2,068 traits, covering a deep catalog of phenotypes extracted from EHR-derived diagnosis codes, clinical laboratory tests, vital signs, and survey responses. The analysis was performed using data from 635,969 participants from MVP Genomics Release 4 classified into four population groups based on genetic similarity (GIA) to the 1000 Genomes Project (Auton *et al.*, 2015; National Academies of Sciences *et al.*, 2023) African (AFR, n = 121,177), Admixed Americans (AMR, n = 59,048), East Asian (EAS, n = 6,702), and European (EUR, n = 449,042) superpopulations. After imputation and quality control (QC) filtering, > 44.3M variants (minor allele count (MAC) ≥ 40) were included for analysis. After trait quality control, 1,854 binary and 214 quantitative traits were included in the downstream analysis in at least one population group.


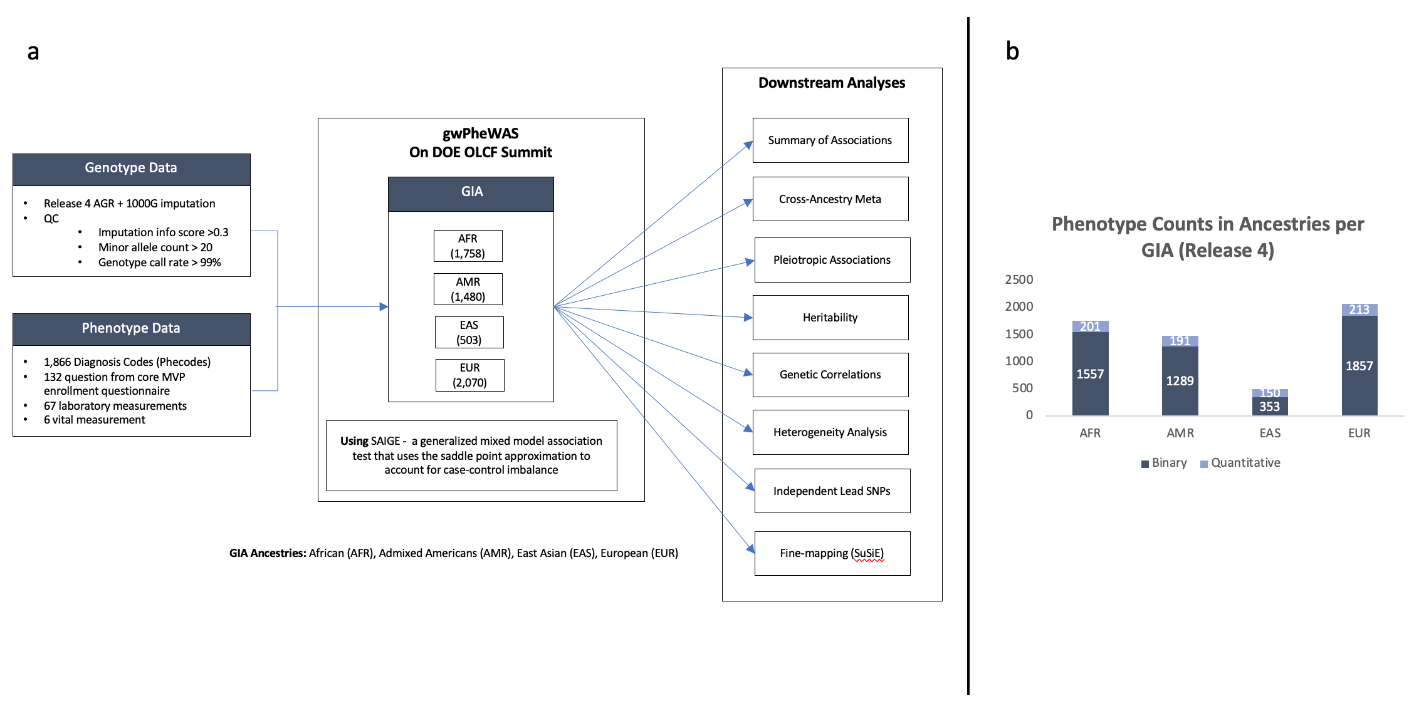


**Supplementary Figure 1** Overview of genomic analysis in multiple population groups. a) Schematic representation illustrating the diverse set of GIA population groups. The analysis covers a deep catalog of traits extracted from electronic health records, clinical laboratory tests, vital signs, and survey responses. b) Chart categorizing traits into binary or quantitative types across different population groups. The height of each bar corresponds to the number of traits in each category, providing an overview of the trait composition for subsequent genomic analyses.

# **Supplementary Figure 2**

To evaluate SAIGE-GPU performance against the native implementation of SAIGE, we deployed both on OLCF’s Frontier System. This test used a containerized-version of native SAIGE v.1.4.4.1 and a simulated dataset of 400,000 individuals of African ancestry. The GPU-version leveraged 3 64-GB GPUs out of 8 available on each node, so 2 traits were able to run in parallel on each node, and 2 GPUs were unused. The native CPU version leveraged all 56 available cores per node.


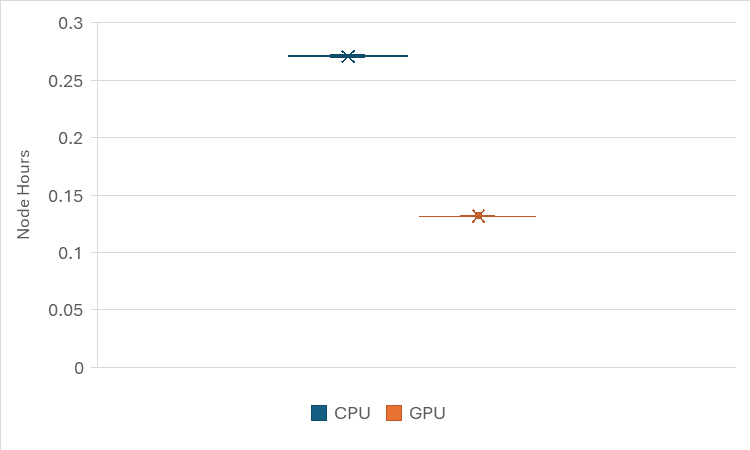


**Supplementary Figure 2** Box plot of SAIGE step 1 performance on 10 traits from a simulated data set of 400,000 individuals. Times plotted are calculated in node hours assuming the GPU version runs two traits per node using 3 GPUs each (6 of 8 available logical GPUs utilized), and the CPU version runs only one trait per node using 56 cores. This allocation allows fair comparison between diverse GPU architectures.

# **Supplementary Figure 3**

To evaluate SAIGE-GPU performance in a cloud environment, we conducted a comparative study that pitted SAIGE-GPU against SAIGE-CPU using data from the UK and AoU Biobanks. We employed the Type 2 Diabetes (T2D) trait to assess their precision, processing speed, and cost-effectiveness within the GCP cloud environment for two of the largest genetically inferred population groups, namely African and European. A 5-fold improvement in execution time was seen from AoU across the European population group (N = 133,000; M = 100,000). Step one completed in 10 minutes using 1 GPU (A100 GPU, 85 GB RAM), whereas the CPU-based SAIGE version consumed 45 minutes on a 64-core virtual machine. Furthermore, the cost of utilizing 1 GPU for the EUR cohort amounted to approximately $0.42, while the cost of the 64-core VM was $3.17. A similar trend in terms of cost and time is observed for the AFR population group, which would have a smaller memory footprint due to the matrix size. This same pattern of advantages is evident when applied to UKBB traits. Specifically, we focused on the EUR population group, which consisted of 420,500 individuals, closely resembling the MVP EUR cohort in participant size. GCP infrastructure (NVIDIA Tesla A100 GPUs, 12 vCPUs, and 85GB of RAM) was employed and completed in just over 30 minutes, with an average cost of $1.45. In contrast, utilizing the CPU-based SAIGE version consumed 58 minutes and incurred a cost of $3.88 using a 96-core VM.


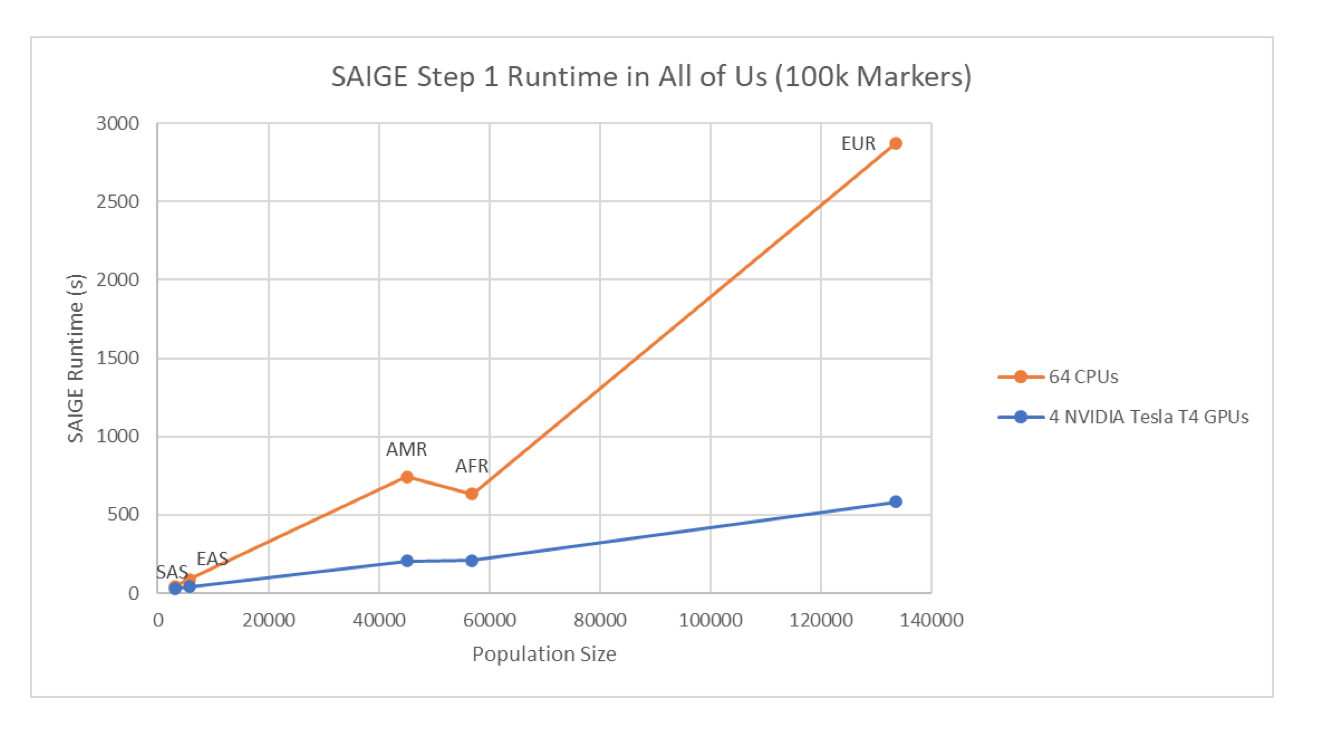


**Supplementary Figure 3** SAIGE step one run time for All of Us data. The figure shows the time comparison of running SAIGE step one for the T2D phenotype on the Google Cloud Platform for the 5 population groups (EUR, AFR, AMR, EAS, SAS). The analysis was executed on 4 NVIDIA T4 GPUs for the SAIGE-GPU version and a 64-CPU VM for the SAIGE-CPU version.

# **Supplementary Table 1**

**Study Design, Population Groups, and Phenotypic Definitions**

The analysis involved a series of GWAS across 2,068 traits, covering a deep catalog of phenotypes extracted from EHR-derived diagnosis codes, clinical laboratory tests, vital signs, and survey responses. The analysis was performed using data from 635,969 participants from MVP Genomics Release 4 classified into four population groups based on genetic similarity (GIA) to the 1000 Genomes Project (National Academies of Sciences *et al.*, 2023; Auton *et al.*, 2015) African (AFR, n = 121,177), Admixed Americans (AMR, n = 59,048), East Asian (EAS, n = 6,702), and European (EUR, n = 449,042) superpopulations.

| **Population Group** | **Participants**  **(Release 4)** |
| --- | --- |
| AFR | 121,177 |
| AMR | 59,048 |
| EAS | 6,702 |
| EUR | 449,042 |

**Supplementary Table 1** Participant quantity in each grouping method per population group. Data was made available on OLCF Summit HPC to perform a GWAS analysis for all traits analysis and all population groups.

# **Supplementary Table 2**

To deal with the large memory requirement, SAIGE relied on the Intel TBB package to parallelize this step, which was incompatible with the Summit infrastructure. We initially replaced the TBB's parallelization method with OpenMP for executing the matrix-vector operations. However, the primary benefit of accelerating step one lies in the considerably faster matrix computations achieved using GPUs compared to CPUs. We compared the SAIGE version that leveraged OpenMP API for parallelization with the GPU version using the Varicose Veins trait (454.1 ICD-9 code). In the OpenMP version, we utilized all 42 available cores on the compute node for parallelizing the matrix calculations to generate the GRM, while for the GPU version we utilized 16 GPUs each equipped with 16 GB of memory in each GPU to distribute subsections of the matrix with dimensions of 8,256 by 445,444. On average, a single PCG iteration on a GPU required approximately 0.069 seconds for the group similar to 1KG-Europe in MVP. In contrast, the OpenMP SAIGE implementation took roughly 5.06 seconds, marking a substantial 72-fold improvement for PCG iterations to converge.

| **Population Group** | **Subjects** | **Step one for Varicose Veins (hours)** | |
| --- | --- | --- | --- |
|  |  | **SAIGE-OpenMP** | **SAIGE-GPU** |
| AFR | 121,725 | 1.06 | 0.38 |
| AMR | 51,124 | 0.97 | 0.28 |
| EAS | 8,003 | 0.58 | 0.23 |
| EUR | 458,307 | 4.10 | 1.50 |

**Supplementary Table 2** Execution times for SAIGE step one on Varicose Veins (ICD-9 code 454.1) using both versions of the SAIGE algorithm on OLCF’s Summit infrastructure. The nodes contain 42-cores and 16 GPUs with 16 GB of RAM.

# **Supplementary Table 3**

As part of the MVP phenome-wide GWAS, we successfully conducted a total of 4,045 independent GWAS runs. The first step of the analysis was accomplished within 14,283 GPU hours on Oak Ridge Leadership Computing Facility’s (OLCF) Summit HPC, equivalent to 5 days of wall time, resulting in a 34-fold reduction relative to projected core CPU hours. Using all 6 GPUs per node, the GPU optimization of step one enabled the completion of the GWAS analysis for all traits and population groups within 2,381 node hours, representing a 5-fold improvement for step one in comparison to a CPU-based implementation on Summit 42-core nodes.

| Population Group | Trait Quantity | Step One CPU hours  for all traits (Projected) | Step One GPU hours  for all traits (Production) |
| --- | --- | --- | --- |
|  |  | SAIGE-OpenMP | SAIGE-GPU |
| AFR | 1,758 | 78,266 | 1,336 |
| AMR | 1,480 | 60,295 | 411 |
| EAS | 503 | 12,253 | 116 |
| EUR | 2,070 | 330,372 | 12,420 |
| **Total** | 5,811 | 481,186 | 14,283 |

**Supplementary Table 3** Times to complete step one of SAIGE when running GWAS for all traits (5,811) in the phenome-wide GWAS using OpenMP and GPU versions on OLCF’s Summit HPC.

# **Supplementary Table 4**

Comparison of time (elapsed time in seconds) required to run step 2 of the GWAS analysis for varying numbers of synthetic traits, illustrating the execution time for individual and simultaneous multi-trait analyses using multi-core CPU parallelization. The table highlights the scaling behavior and efficiency of running single versus multiple traits on available computational cores. For runs with more than three traits on a single core, the default job wall-time allocation of 2 hours was insufficient. Simulated data consisted of 400,000 samples generated using HapGen2 (Su *et al.*, 2011) and African-ancestry haplotypes for chromosome 1 from the 1000 Genomes Project (Auton *et al.*, 2015). 15 binary traits were simulated using a simple binomial distribution with a case-probability of 50%. There was a 15% computational time saving when running all 15 traits in parallel versus sequentially.

| Trait Description | | Execution Times (seconds) | | |
| --- | --- | --- | --- | --- |
| Trait Quantity | Trait ID | Single-core | 64-cores | 64-cores per trait |
| 1 | 1 | 2,170 | 269 | 269 |
| 1 | 2 | 2,180 | 275 | 275 |
| 1 | 3 | 2,190 | 290 | 290 |
| 1 | 4 | 2,250 | 314 | 314 |
| 1 | 5 | 2,200 | 286 | 286 |
| 1 | 6 | 2,287 | 280 | 280 |
| 1 | 7 | 2,211 | 277 | 277 |
| 1 | 8 | 2,203 | 286 | 286 |
| 1 | 9 | 2,172 | 275 | 275 |
| 1 | 10 | 2,190 | 305 | 305 |
| 1 | 11 | 2,377 | 341 | 341 |
| 1 | 12 | 2,294 | 298 | 298 |
| 1 | 13 | 2,307 | 309 | 309 |
| 1 | 14 | 2,185 | 281 | 281 |
| 1 | 15 | 2,198 | 273 | 273 |
| 2 | 1,2 | 4,205 | 504 | 252 |
| 3 | 1,2,3 | 6,145 | 743 | 248 |
| 10 | 1-10 | > 2 hours | 2,526 | 253 |
| 15 | 1-15 | > 2 hours | 3,720 | 248 |

**Supplementary Table 4** Times to complete step two of SAIGE on a simulated data set of 400,000 individuals of AFR Ancestry and 15 binary traits.

# **Supplementary Table 5**

Comparison of total computational time (elapsed time in seconds) required to run step 2 of the GWAS analysis for 15 synthetic traits with native SAIGE and SAIGE-GPU. SAIGE-GPU ran all 15 traits simultaneously while native SAIGE ran all 15 in sequence. Across all chromosomes there was an 11.5% improvement in time for the parallel computation.

| Chromosome | SAIGE-GPU | Native-SAIGE | Difference |
| --- | --- | --- | --- |
| chr1 | 4574 | 4429 | 145 |
| chr2 | 4832 | 5041 | -209 |
| chr3 | 4122 | 4382 | -260 |
| chr4 | 3887 | 3999 | -112 |
| chr5 | 3576 | 3853 | -277 |
| chr6 | 3945 | 4187 | -241 |
| chr7 | 3354 | 3566 | -212 |
| chr8 | 3177 | 3404 | -227 |
| chr9 | 2716 | 3124 | -408 |
| chr10 | 3105 | 3656 | -550 |
| chr11 | 3094 | 3853 | -759 |
| chr12 | 2947 | 3809 | -861 |
| chr13 | 2378 | 3561 | -1182 |
| chr14 | 2167 | 2503 | -335 |
| chr15 | 2072 | 2269 | -197 |
| chr16 | 2144 | 2426 | -282 |
| chr17 | 1954 | 2286 | -332 |
| chr18 | 2041 | 2362 | -321 |
| chr19 | 1676 | 1892 | -216 |
| chr20 | 1850 | 2154 | -304 |
| chr21 | 1244 | 1755 | -512 |
| chr22 | 1261 | 1651 | -389 |

**Supplementary Table 5** Relative times of native SAIGE and SAIGE-GPU to complete step two on a simulated data set of 400,000 individuals of AFR Ancestry and 15 binary traits.

# **Supplementary Table 6**

To assess whether GPU acceleration addresses a genuine computational bottleneck in GWAS analysis, we conducted direct performance comparisons between SAIGE-GPU and REGENIE (v4.1), one of the leading alternative GWAS methods. Both methods were evaluated on identical datasets and hardware to enable fair comparison. SAIGE and REGENIE employ fundamentally different statistical approaches. REGENIE uses an approximate two-step strategy prioritizing speed; SAIGE uses exact generalized linear mixed model inference. For analyses prioritizing statistical control, precision, and validation of previous findings—particularly in case-control studies with imbalanced phenotypes or investigations of rare variants—SAIGE's exact approach is preferred. For discovery-oriented analyses where approximate methods are acceptable, REGENIE is appropriate. These methods are complementary rather than competitive.

We analyzed a simulated dataset with 400,000 samples and 121,587 variants across 10 binary phenotypes. Both SAIGE-GPU and REGENIE were executed using the same genotype data, covariates (10 principal components), and statistical parameters (Firth regression and saddlepoint approximation for binary phenotypes).

Step 1 Performance (Model Fitting): SAIGE-GPU completed step 1 in approximately 15 minutes using 3 GPUs, while REGENIE required 31-34 minutes using 16-32 optimally-tuned CPU threads (Supplementary Table 1). This represents a 2-2.3× speedup for SAIGE-GPU's GPU-accelerated implementation.

| Method | Version | Hardware | Dataset | Step 1 Time | Speedup |
| --- | --- | --- | --- | --- | --- |
| SAIGE-GPU | v1.3.3 | 3x MI250X GPUs | 400k samples, 121k variants | ~15 min | 2-2.3x |
| REGENIE | v4.1 | 16-32 CPU threads | 400k samples, 121k variants | 31-34 min | baseline |

**Supplementary Table 6** Comparison focuses on Step 1 (model fitting), which represents the primary computational bottleneck in SAIGE's exact approach. Both methods use the same phenotype data, covariates (10 PCs), and statistical parameters (Firth regression + SPA).

**Supplementary Table 7**

REGENIE requires careful parameter tuning (thread count and block size) for optimal performance. SAIGE-GPU demonstrates more robust scaling across GPU counts without requiring parameter optimization. Benchmarking REGENIE revealed important differences in optimization requirements. REGENIE's approximate approach requires careful tuning of computational parameters (thread count and block size). Single-threaded REGENIE exceeded 120 minutes, with optimal performance at 16 threads and block size 1000 (31 minutes). Further parallelization showed diminishing returns (32 threads: 33 minutes, 127 threads: >120 minutes). In contrast, SAIGE-GPU demonstrates more robust scaling across GPU counts (3, 6, 8 GPUs) without requiring parameter tuning, highlighting differences between approximate and exact method optimization strategies.

SAIGE's exact generalized linear mixed model approach concentrates most of the computational cost in step 1 (solving iterative linear systems via preconditioned conjugate gradient). Each PCG iteration demands numerous matrix-vector multiplications with the genetic relationship matrix—operations with regular structure and high arithmetic intensity, ideally suited to GPU execution.

| Cores | REGENIE Block size | Execution Time (minutes) | Core hours | Notes |
| --- | --- | --- | --- | --- |
| 1 | 1000 | >120 | >2 | Exceeds 2-hour HPC limit |
| 8 | 1000 | 34 | 4.5 | Modest parallelization |
| 16 | 100 | >120 | >32 | Memory thrashing |
| 16 | 1000 | 31 | 8.2 | Optimal performance |
| 16 | 5000 | >120 | >32 | Memory issues |
| 32 | 1000 | 33 | 17.6 | Diminishing returns |
| 32 | 5000 | >120 | >64 | Memory issues |
| 127 | 1000 | >120 | >254 | Over-parallelization |

**Supplementary Table 7** Benchmark results for running REGENIE model fitting to fine-tune the parameter utilization.

# **References**

Auton,A. *et al.* (2015) A global reference for human genetic variation. *Nature*, **526**, 68–74.

National Academies of Sciences,E. *et al.* (2023) COMMITTEE ON THE USE OF RACE, ETHNICITY, AND ANCESTRY AS POPULATION DESCRIPTORS IN GENOMICS RESEARCH. In, *Using Population Descriptors in Genetics and Genomics Research: A New Framework for an Evolving Field*. National Academies Press (US).

Su,Z. *et al.* (2011) HAPGEN2: simulation of multiple disease SNPs. *Bioinformatics*, **27**, 2304–2305.
